# Targeting intracellular galectins for cancer treatment

**DOI:** 10.3389/fimmu.2023.1269391

**Published:** 2023-09-11

**Authors:** Rita Nehmé, Yves St-Pierre

**Affiliations:** INRS-Centre Armand-Frappier Santé Biotechnologie, Laval, QC, Canada

**Keywords:** cancer, galectin, intracellular, inhibitors, nanobodies, intrabodies

## Abstract

Although considerable attention has been paid to the role of extracellular galectins in modulating, positively or negatively, tumor growth and metastasis, we have witnessed a growing interest in the role of intracellular galectins in response to their environment. This is not surprising as many galectins preferentially exist in cytosolic and nuclear compartments, which is consistent with the fact that they are exported outside the cells via a yet undefined non-classical mechanism. This review summarizes our most recent knowledge of their intracellular functions in cancer cells and provides some directions for future strategies to inhibit their role in cancer progression.

## 1 Introduction

Inside the cells, galectins are best known to form aggregates following the recognition of glycans on the surface of damaged endocytic vesicles, including damaged phagosomes, endosomes, and lysosomes (1). Although galectins bind carbohydrates, it is also becoming apparent that intracellular galectins are involved in carbohydrate-independent interactions with multiple ligands. This may not be surprising as some galectins harbor distinctive glycan binding sites (GBS) that preclude binding to β-galactoside and other carbohydrates (2–4). For example, galectin-10, also known as the Charcot-Leyden crystals (CLC), binds in a carbohydrate-independent manner with intracellular RNases, modulating their translocation inside eosinophils (5). In the case of galectin-16, it binds via protein-protein interactions to c-Rel, an NF-kB subunit known to play a central role in multiple types of cancer (4). The ability of intracellular galectins to accomplish various functions via protein-protein interactions is not restricted to galectins with limited carbohydrate binding functions. Still, it is shared by multiple, if not all, galectin family members. These protein-protein interactions control many cellular processes linked to cancer progression, such as resistance to drug-induced apoptosis, cell transformation, expression of cancer genes, proliferation, dysregulation of cellular metabolism or cytoskeletal remodeling, obliging us to rethink our strategies to design galectin drugs in the context of specific diseases. Here, we discuss the challenges and opportunities associated with targeting intracellular galectins for cancer treatment.

## 2 Where are galectins in cancer cells?

The short answer to this question is “almost everywhere”. We find galectins in the cytosol, the nucleus, or associated with subcellular organelles, such as mitochondria or endocytic vesicles. This has been well documented for those galectins such as galectin-1, -3, and -9 (6). The movement of galectins will be decisive for the cell’s survival, particularly its resistance to apoptosis and the acquisition of an invasive phenotype. The recent literature has shown, however, that this paradigm also applies to many, if not all, other galectins. A case in point is galectin-8, which has attracted the attention of many researchers of different disciplines following the discovery that intracellular galectin-8 acts as a danger signal following the recognition of pathogen-damaged endocytic vesicles in the cytosol (1). Recent studies have also shown that intracellular galectin-8 and galectin-9 control cellular metabolism and autophagy by modulating the activation state of the Ser/Thr protein kinase MTOR (mechanistic target of rapamycin kinase) and AMPK (5’ AMP-activated protein kinase) (7, 8). Whether intracellular galectin-8 controls cancer progression is not clear yet, but the fact that it controls key signaling pathways suggests that it may be the case in several types of cancer (9). Galectin-8 might also play a central role in cell proliferation. This hypothesis is supported by the study of Lo and colleagues, who showed that galectin-8 is localized to the mitotic apparatus and associated with centrosomes, possibly contributing to the organization of the mitotic structure and the regulation of cell-cycle progression (10). As in the case of other galectins, however, galectin-8 may have a dual role depending on the cancer subtype or its intracellular localization. Subcellular localization of galectins in cancer cells is an important yet often underestimated issue considering that there is a considerable amount of literature showing that the functions of galectins inside depend on their subcellular localization (11–16). A good example is the expression of galectin-1 and galectin-8 in cancer cells. In patients with triple-negative breast cancer (TNBC), an aggressive subtype of breast cancer which lacks HER2, estrogen and progesterone receptors, the presence of galectin-8 in the nucleus is associated with good disease-free (17). In contrast, high expression of nuclear galectin-1 correlates with a bad prognosis and overall survival. Interestingly, patients who were positive for nuclear galectin-1 and galectin-8 were found to have a 5-year disease-free survival of 100%, indicating that galectin-8 impacted on the role of galectin-1. Such distinctive intracellular distribution of galectin-8 has also been observed in colon cancer by Nagy and colleagues, who found that while galectin-8 was located in both the cytoplasm and nuclei of normal and benign colon tissue, it was located exclusively in the cytoplasm of malignant colon cells (18). This dual localization of galectin-8 has also been observed in Warthin’s tumor, a benign neoplasm of the salivary gland (19, 20). In contrast, a recent study in patients with cervical cancer revealed that galectin-8 was only expressed in the cytoplasm but not in the nucleus, suggesting that localization of galectin-8 can be localized to a specific compartment, depending of the tumor stage and the cancer type (21). This exclusive cytosolic localization was also observed in the case of galectin-9. Interestingly, expression of both galectin-8 and -9 was associated with relapse-free survival and a better prognosis regarding overall survival, respectively. Such an association between low galectin-8 expression and improved survival has also been observed by Trebo and colleagues (22). This is a clear contrast with the expression of galectin-7 in breast cancer, where its expression not only correlates with cancer progression but also promotes metastasis (23).

Another interesting case that has emerged recently is galectin-4, which expression has been primarily studied in epithelial cells of the gastrointestinal tract. Although the number of studies on the role of galectin-4 in cancer remains relatively low (approximately 60 studies in the last ten years, compared to more than a thousand for galectin-3, for example), we are starting to get a better view of its possible implication in cancer, not only outside the cells but also inside, allowing us to pinpoint its role in cancer progression (24–27). In the case of nasal papilloma and squamous carcinoma, for example, Duray and colleagues showed that galectin-4 can be expressed in both cytosolic and nuclear compartments but sometimes only in a single compartment (20). Similar results were obtained in lung, pancreatic, ovarian and hepatocellular carcinomas (28–33). In many cases, the authors observed that expression of galectin-4 was downregulated in patients with advanced stages of the disease and patients with good survival, leading to the hypothesis that galectin-4 may act as a tumor suppressor, at least for specific types of cancer (34). The current view is that galectin-4 inside the cells interferes with the Wnt signaling pathways and inhibits cell proliferation and migration of colorectal cancer cells (30). This does not apply, however, to all cancer subtypes. Suppression of galectin-4 expression in gastric cancer has recently been shown to reduce metastasis (35). The development of an elegant HCT-116 colon cell model where expression of the gene encoding human galectin-4 is regulated by doxycycline will likely contribute significantly to improving our understanding of the role of galectin-4 in cancer progression (36). It might help to understand better, for example, the molecular mechanism regulating compartmentalization, a dynamic process regulated upon binding to intracellular ligands, including chaperones.

With respect to other human galectins, including galectin-2, -10, -12, -13, -14 and -16, our knowledge of these galectins remains rather fragmentary regarding their intracellular roles in cancer, or even their role once released into the tumor microenvironment for that matter. It is reasonable to believe that the accessibility of new tools for detecting their protein expression and modulating their activity will contribute to a better understanding of their role in tumor progression. This is particularly the case for placental galectins (galectin-13, -14, and 16), a subgroup whose biological characteristics share many points with the functions associated with cancer development (37, 38). Concerning galectin-2, a recent study demonstrated that it plays a crucial role in TNBC by contributing to the formation of an immunosuppressive microenvironment, possibly following the release of its form extracellular by the tumor cell (39). These observations add to prior studies establishing galectins as prime targets for improving the efficacy of immunotherapy within the tumor microenvironment (40–42). Galectin-2 has also been found in the cytosol and nuclei of gastric cancer cells (43). In this case, Jung and colleagues reported that loss of galectin-2 in gastric cancer cells was associated with the aggressiveness of cancer cells, revealing a commonality between galectin-2 and other galectins in playing contradictory roles in tumor progression, depending on the type. Regarding galectin-10, the galectin that forms CLC in eosinophils, its role in cancer has not been highly investigated. However, we do know that it is expressed in the cytosol of ovarian cancer cells and, like galectin-4 and galectin-13, is possibly associated with a favorable prognosis (44). But just like these other less well-known galectins, their intracellular role and the nature of their intracellular interactions in various types of tumor cells remain unknown.

## 3 What are the binding partners of galectins inside cancer cells?

**Figure 1:**
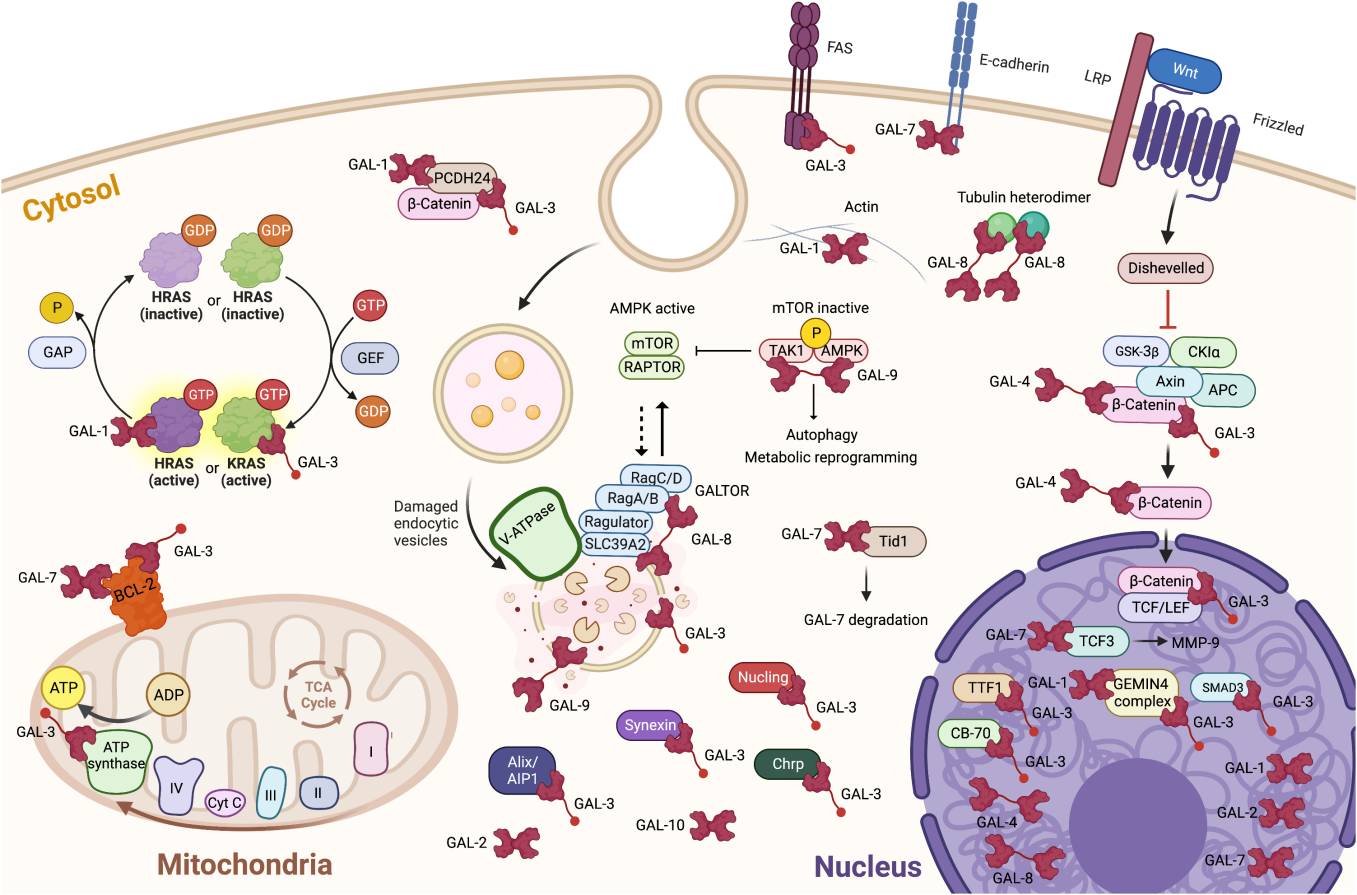
Localization and binding partners of intracellular galectins in cancer cells. It is also important to take into account that, in most cases, we still ignore the intracellular ligands of galectins. The knowledge of the target site(s) remains also very fragmentary. It is thus important to consider that two galectins may compete for the same binding site on any given ligand(s). Created with BioRender.com.

The importance of intracellular galectins in cancer progression goes back decades ago but attracted the attention of many when it was shown that galectin-1 binds activated H-Ras, stabilizing its anchorage to the cell membrane (45). This study was undoubtedly a turning point in recognizing the importance of intracellular galectins in tumor progression. Since then, much water has flowed under the bridge, making it possible to understand better the importance of the interactions between galectins and their intracellular ligands, particularly in response to signals originating from the tumor microenvironment. Nowadays, direct interactions between galectins and their ligands have been characterized, revealing dependent or not on their sugar-binding activity (**Figure 1**). In the case of galectin-3, this includes proteins such as β-catenin, hnRNPA2B1, gemin4, nucleoporin 98, importin-α, and Alix, a component of the endosomal sorting complex required for intracellular transport (16, 46–49). In the case of Alix, this involves a sugar-independent mechanism (47). For galectin-7, this includes sugar-independent binding to bcl-2 and E-cadherin (1, 14). Galectin-7 also binds, in a carbohydrate-dependent manner, to human tumorous imaginal disc (Tid1) heat shock protein 40 (Hsp40), and this interaction attenuates tumorigenicity and metastasis of head and neck squamous sarcoma cancer cells (50). Tid1 seems to prevent the translocation of galectin-7 to the nucleus, suppressing galectin-7 protumoral activity. These results support the previous hypothesis that Tid1 is a tumor suppressor in head and neck squamous cell carcinoma (47). They also provide a possible explanation for the previous finding that a mutation in the glycan binding site of galectin-7 promotes cancer progression compared to wild-type galectin-7 (51). Future research is needed to characterize the interaction between galectins and Tid1 better and to determine whether this interaction could explain the dual role of galectins in cancer. This is an important question, as cancer cells express more than one intracellular galectin (17, 33).

Notwithstanding the nature and type of interaction between intracellular galectins and their ligands, a trend emerges that these interactions modulate, positively or negatively, signaling pathways that come together in response to the tumor microenvironment. This new paradigm derives from studies using cancer models and studies highlighting cytosolic galectins’ role as danger sensors during membrane damage induced by infectious agents (1). This has been particularly well established in the case of galectin-8 and its ability to modulate the mTOR pathway. GST-pulldown assays in HeLa cells revealed a direct interaction between galectin-8, but not galectin-9, and all four Rag GTPases. This interaction requires a full-size galectin-8 and a functional CRD2 domain, suggesting it involves carbohydrate-dependent binding (7). The mTOR protein is the catalytic subunit of two distinct protein complexes, mTOR complex 1 (mTORC1) and mTOR complex 2 (mTORC2). In cancer cells, the two complexes are critical regulators in the signal transduction networks that control several cell functions, including cell division and survival (52). It is, therefore, logical to raise the hypothesis that galectin-8 can act as a tumor suppressor via its “GALTOR”-like activity, a term used by Jia and collaborators to define a dynamic galectin-based regulatory subsystem controlling mTOR (7).

In summary, galectins undergo a complex intracellular journey in cancer cells through several compartments in response to environmental signals. Through this pathway, they bind to multiple ligands involved in trafficking and cell activation, and this via sugar-dependent and independent interactions. What is the degree of redundancy between these interactions for a cell that sometimes expresses a varied repertoire of intracellular galectins remains an open question that will eventually need to be answered to fully exploit galectins’ potential as therapeutic targets.

## 4 Targeting intracellular galectins

**Figure 2:**
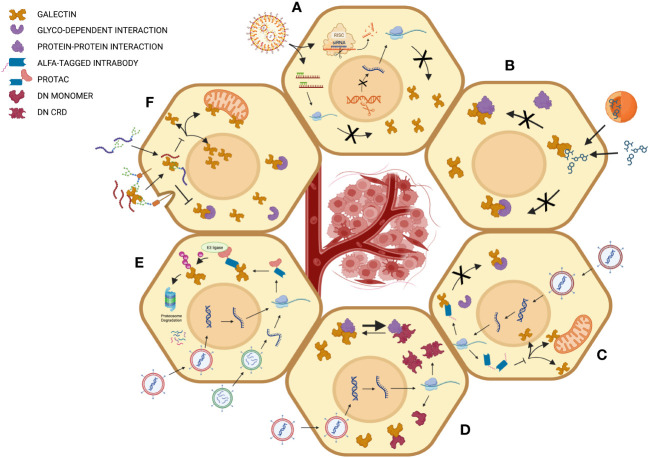
Different strategies for intracellular targeting of galectins in cancer cells. **(A)** Inhibition by gene silencing using RNA interference, antisense strategies or CRISPR-mediated gene knockdown for inhibition of galectin genes at the transcriptional or post-transcriptional levels. **(B)** Inhibition of intracellular glycan-dependent interaction using carbohydrate-based inhibitors or other small drugs that modulate the binding of glycosylated intracellular ligands. The mechanism of action may imply direct binding to the glycan-binding site of galectins or an allosteric effect that modulates the activity of the GBS. **(C)** Inhibition using intrabodies. Such a strategy may inhibit galectin’s intracellular activity via multiple mechanisms of action, including inhibition of both carbohydrate-dependent and independent interactions and modulating intracellular trafficking of galectins. **(D)** The expression of dominant negative (DN) mutants can be used to interfere with the interaction of galectins to different types of ligands or simply by interfering with the formation of homodimers in the case of prototypic galectins. **(E)** This strategy employs proteolysis-targeting chimeras (PROTACs), which bring galectins to the ubiquitination machinery. In this case, the intrabody can be linked, for example, to a subunit of the Von Hippel–Lindau (VHL) with ubiquitin E3 ligase activity, a common strategy to target intracellular proteins of interest. **(F)** Peptide-mediated inhibition is mediated using either glycopeptides or following receptor-mediated entry of peptides. Created with BioRender.com.

Although the number of galectin inhibitors having reached clinical trials for the treatment of cancer remains relatively modest, it is surely not because of the lack of effort. The development of galectin inhibitors faces several major challenges, if only the difficulties encountered at the conceptual level, such as their sometimes contradictory role in tumor progression, and at the structural level, considering the significant homology between the different members of galectins and the redundancy of their functions. In this context, several strategies have been adopted or are being developed, several of which are perfectly suited for the inhibition of their role at the intracellular level (**Figure 2**).

### 4.1 Small pharmacological inhibitors

There are several challenges associated with small pharmacological inhibitors being effective intracellularly. The first is the need to cross the plasma membrane. A recent study by Stegmayr and collaborators has compared the cellular uptake of three high-affinity (in the low nanomolar range) galectin-3 thiodigalactosides and α-d-galactoside inhibitors (53). Using JIMT-1 breast cancer cells, the authors reported striking differences in the cellular uptake of the inhibitors and their ability to reduce the accumulation of galectin-3 around chemically-induced disruption of intracellular vesicles of JIMT-1 breast cancer cells. A similar approach but in a different type of breast cancer cells (MCF-7) was used to test the cell permeability of other synthetic small-molecule galectin inhibitors (54). The authors reported that one of their inhibitors blocked amitriptyline-induced vesicle damage in breast carcinoma MCF-7 cells, possibly by blocking the interaction between galectin-3 and LAMP1/2 proteins. Such studies with *in vitro* cell systems can clearly help to define which properties are needed to obtain better cell permeability and to target intracellular galectins for specific cell types. Yet, the number of studies looking at inhibiting the intracellular functions of galectins with small pharmacological inhibitors remains relatively rare as most of these inhibitors target extracellular galectins. Moreover, although some of these inhibitors can cross the cell membrane, it remains unclear if they can induce long-lasting inhibition, impact protein-protein intracellular interactions, or act on other intracellular galectins. Thus, although several studies have shown excellent permeability for some synthetic small-molecule galectin inhibitors, it is imperative to determine if they can reach the galectin pools and inhibit specific interactions with galectin’s ligands. This is not a trivial task, as galectins may be located in distinct intracellular compartments, including the nucleus. This is why it is crucial to determine where the protumorigenic roles of galectins are expressed. A third challenge is identifying the intracellular ligand(s) of interest and the functional target site. Many small pharmacological inhibitors were designed to target the glycan-binding site of galectins. Whether these GBS-specific inhibitors can interfere with the protein-protein interactions of galectins is a real possibility, as binding of a ligand within the GBS can affect the overall structure of the CRD via allosteric mechanisms (55). Another challenge is to achieve specificity. There is, however, a reason to be optimistic in this regard, as differences between galectin binding sites allow a fine-tuning of ligand selectivity and potency, a strategy that has been successfully exploited in the past by the group of Nilsson and colleagues and more recently for the generation of galectin-8-specific inhibitors (56). Yet, it might be profitable to inhibit multiple galectins in a cancer cell if, and only if, these galectins are all pulling the wagon in the same direction. Thus, although these obstacles may seem difficult to overcome, they can nevertheless be circumvented by adapting drug screens at the earliest stages of development.

### 4.2 Antisense-oligonucleotide and siRNA drugs

Genetic silencing using antisense oligonucleotides (ASOs) and siRNAs has been the cornerstone of fundamental research on the role of galectin in cancer progression. This is mainly because specificity is theoretically easier to achieve and the absence, or relative rarity, of highly specific research tools. Knockdown of galectins using ASOs has been extensively used to demonstrate the role of galectin-1 and galectin-3 in cancer (57–60). It has also been used for less well-known galectins or when manipulating galectin gene expression in model organisms, such as Zebra fish (61). We and others have also used this approach to establish the role of galectin-7 in cancer progression (62) or the role of galectin-4 (63). ASO drugs have been an excellent research tool given their ease of production and versatility, considering the structural homology between galectins expressed in the same cancer cell. In recent years, ASOs have been replaced by siRNA-based drugs for galectin research. In most cases, siRNAs have been used with *in vitro* cell models or for measuring the effect of the knockdown of a specific galectin in its tumorigenic potential once implanted in mice. For example, in a recent report, Li and colleagues used galectin-9-specific siRNA loaded into exosomes decorated with transferrin receptor-binding peptides to target glioblastoma cells and inhibit their growth, opening the way to novel immunotherapies of glioblastoma (64). Orozco and colleagues have also shown that it is possible to use siRNA to deplete galectin-1 in pancreatic stellate cells, which inhibited cancer progression and metastasis when co-injected with pancreatic tumor cells (65). However, using siRNA-loaded nanocarriers to inhibit galectin expression *in vivo* remains rare but clearly possible. Using a mouse preclinical pancreatic cancer model, Zhou and colleagues have shown that it is possible to inhibit galectin-9 expression in the host following the intravenous injection of galectin-9-specific siRNA-loaded exosomes (66). When combined with oxaliplatin, the authors observed a quite remarkable reduction in the growth of the primary tumor and a significant increase in the overall survival when compared to control groups. Before bringing such drugs to patients, however, several hurdles still need to be overcome, explaining at least in part why there are still very few FDA-approved siRNA drugs, which are generally reserved for undruggable targets or genetic diseases, particularly those caused by protein overexpression. The site-specific delivery and rapid clearance are the main challenge in using siRNA-based drugs. Yet, the number of clinical trials testing siRNA therapeutics is on the rise following significant development in strategies to improve siRNA delivery for patients with solid tumors, especially for cancers with limited treatment options, such as pancreatic cancer.

### 4.3 Using peptides

The use of peptides for inhibiting galectins has received much attention with the development of Anginex and its derivatives. However, it didn’t exactly lead to the expected clinical success, primarily for reasons related to their pharmacological properties (6). In addition, we still need to learn more about its specificity. We know that it binds to galectin-1 and -3, but little is known about its ability to bind to other galectins and murine forms, important information for preclinical studies in animal models. Moreover, although this peptide has generated encouraging results in its ability to reduce angiogenesis in pre-preclinical models, it needs to be clarified whether this effect depends on its interaction with galectin-1 or galectin-3. That being said, there is every reason to believe the development of new high-throughput screening strategies will make it possible to generate highly specific peptides with optimal pharmacological properties to inhibit galectins. And all the more so since we have witnessed in recent years significant advances in our knowledge of the mechanisms of transport and translocation of peptides in the cytosol (67, 68). Our group has also demonstrated that it is possible to generate galectin inhibitor peptides that are highly specific and capable of inhibiting both sugar-dependent and sugar-independent interactions (69, 70). These peptides, called DIPs (Dimer-Interfering Peptides), were developed by rational design following a careful analysis of the interface of galectin-7 homodimers, which offers an exciting source of specificity for the development of galectin inhibitors. The binding of these peptides at the monomer interface inhibits the formation of functional dimers, making this mechanism of action well-suited for inhibiting prototypical galectins.

Many others have also identified galectin-specific peptides using different strategies, such as the screen of random phage libraries. One of the peptides, G3-C12, binds human galectin-3 with relatively high (70 nM) affinity (71). This peptide inhibits the interaction of galectin-3 to carbohydrate Thomsen**-**Friedenreich tumor antigen, a galactose β1-3*N*-acetylgalactosamine disaccharide. The authors subsequently showed that G3-C12 significantly reduced the growth of human MDA-MB-231 breast carcinoma cells in nude mice (71). However, it was unclear whether this *in vivo* effect was galectin-3-dependent, and if so, whether the therapeutic effect resulted from the interaction of the peptide with human galectin-3 or mouse galectin-3 secreted by the host in the tumor microenvironment. In a “Two Birds, One Stone” strategy, Sun and colleagues used the G3-C12 peptide embedded in a polymer to disrupt mitochondrial functions of prostate cancer cells following its entry into the cytosol via a receptor-mediated mechanism or to facilitate the entry of the anti-tumor drug 5-fluorouracil (72, 73). This study further demonstrated that it is possible to use galectin-specific ligands combined with specific delivery systems to inhibit a galectin’s extracellular function and deliver intracellular therapeutic loads inside the cells (74).

A variation of the peptide strategy is to use dominant-negative peptides or protein fragments, a strategy commonly used as a research tool to interfere with intracellular signaling functions in cell biology. The group of Ron Patterson has used this strategy to inhibit galectin-3’s function of splicing pre-mRNA in a cell-free splicing assay (75). They used the N-terminal polypeptide domain of galectin-3 to inhibit the spliceosome formation. Today, it is possible to use high-throughput methods to identify dominant negative gene fragments that are specific for a protein or its specific subdomain(s), including sequencing of DNA libraries encoding *S. cerevisiae* polypeptides or lentiviral overexpression libraries of peptides (76, 77). In summary, it is logical to foresee that peptide-based strategies could be adapted for other galectins, considering the molecular interactions between the monomers of tandem repeat-type galectins.

### 4.4 Using intrabodies

Over 30 years ago, camelid antibodies were discovered and completely transformed how we understood the structure of antibodies (78). New technological platforms have since been developed to utilize the distinct characteristics of camelid antibodies, offering exciting opportunities to use their exceptional properties, including their ability to accurately bind a diverse range of antigens with high specificity and affinity. These antibodies often referred to as nanobodies (Nbs), which is a trademark of Ablynx, are different from traditional antibodies in that they can bind to antigenic epitopes more effectively due to a single, variable domain encoded in the heavy chain fragment (VHH). Another unique feature of Nbs is their extended convex-shaped paratope, which can recognize epitopes that are usually inaccessible to conventional antibodies. This is possible because their hypervariable region is made of a single stretch of amino acid residues composed of flexible peptide loops, including a relatively long complementary determining region (CDR)-3 loop that is extended and made of 15-25 residues on average (compared to 12 residues in human) (79). These properties have been exploited to generate, for example, Nbs capable of recognizing hitherto inaccessible antigenic epitopes of catalytic sites of enzymes, such as matrix metalloproteases (MMPs), a family of enzymes with minimal active site specificity (80). The specificity of MMP inhibitors has always been a significant problem, given the contradictory roles of MMPs during cancer progression (81). Like MMPs, galectins have stage- and tumor-specific roles, even contradictory (dual) roles. Single-chain Nbs are among the new generation of MMP inhibitors that have contributed to the rebirth of the interest in MMPs as anti-cancer drugs in clinical trials (82). Such success could reflect in the development of galectin-specific Nbs whose antigen-binding region’s structure is perfectly designed to target epitopes located at the dimer interface of prototypic galectins or those buried deep within the GBS. Yet, although Nbs are increasingly used for therapeutic purposes, it is only in recent years that we have witnessed their use to target intracellular proteins, leading to the concept of “intrabodies” (83–85). A simple strategy when using intrabodies is to use Nbs with an ALFA tag, a compact short hydrophilic, uncharged 15 amino acid sequence that readily adopts an alpha-helix structure that spontaneously refolds even after exposure to harsh chemical treatment (such as those used to fix cells or during SDS-PAGE). This tag is functional irrespective of its position on the target protein and developed by rational design for the labelling of Nbs (86). The rationale behind using ALFA-tagged Nbs is that they can potentially interfere with intracellular protein-protein interactions essential for galectins to exert their protumorigenic functions. It is also likely that such intrabodies will interfere with the intracellular localization of galectins by interfering with binding partners acting as chaperones. This is an important issue as the role of a given galectin in cancer may well depend on its intracellular distribution. Such intrabody would thus not only allow the development of novel therapeutics but also provide research tools for investigating the importance of subcellular localization with cancer progression.

Another interesting option offered by Nbs is to use proteolysis-targeting chimeras (PROTACs) that will bring the nanobody-galectin complex to the ubiquitination machinery, leading to polyubiquitination of the galectin and subsequent proteasomal degradation (87). Often referred to as an “*affinity-directed protein missile system,”* this strategy is increasingly used to obtain sustained inhibition of an intracellular protein expression (88–90). It is logical to predict that PROTACs would reduce the intracellular pool of galectins and the release of extracellular galectins, limiting their ability to create local immunosuppression via the killing of activated immune cells or neutralizing cytokines that are essential for the recruitment and activation of immune cells. This “two birds with one stone” strategy could be an interesting approach when combined with immune checkpoint inhibitors as it would help the patients to mount a cancer-specific immune response. On a long-term basis, we can envision the delivery of mRNA-encoding galectin-specific intrabodies to target cancer cells, taking advantage of the immense progress on mRNA vaccines accomplished during the recent pandemic (91–93). These strategies are at our doorstep since several groups have already reported the development of galectin-specific Nbs and their derivatives against galectin-1, -2, -7 and -10 (94–97).

## 5 Summary

Much interest in galectins has been devoted to their extracellular roles, notably in controlling the immune response and their ability to modulate intracellular signaling through their interaction with glycosylated membrane receptors. However, recent studies have demonstrated their ability to interact directly with numerous intracellular ligands in various compartments, making them targets for modulating signaling networks. It is logical to believe that their ability to modulate these networks will directly affect the expression of critical genes in tumor development and perhaps even epigenetic modifications considering their key role in the interface between the cell and its tumor microenvironment, which is highly adaptable and undergo frequent changes that will impact the efficacy of the inhibitors (98). Moreover, surprisingly, few studies have been devoted to the impact of galectins, whether in their intra or extracellular form, on the transcriptome. There is no doubt that intracellular galectins will reveal new surprises and positions for developing new cancer therapies, especially aggressive cancers for which few therapeutic options exist.

## Author contributions

YSP: Conceptualization, Funding acquisition, Supervision, Writing – original draft. RN: Conceptualization, Data curation, Writing – review & editing.
